# A Scoping Review of Sarcoglycan Expression in Non-Muscle Organs: Beyond Muscles

**DOI:** 10.3390/biom15071020

**Published:** 2025-07-15

**Authors:** Fabiana Nicita, Josè Freni, Antonio Centofanti, Angelo Favaloro, Davide Labellarte, Giuseppina Cutroneo, Michele Runci Anastasi, Giovanna Vermiglio

**Affiliations:** 1 Department of Biomedical, Dental Sciences and Morphofunctional Imaging, University of Messina, 98125 Messina, Italy; antonio.centofanti@unime.it (A.C.); angelo.favaloro@unime.it (A.F.); davidelabellarte99@gmail.com (D.L.); giuseppina.cutroneo@unime.it (G.C.); gvermiglio1@unime.it (G.V.); 2 Department of Odontostomatological and Maxillofacial Sciences, “Sapienza” University of Rome, 00185 Rome, Italy; 3 Department of Maxillo-Facial Surgery, “Sapienza” University of Rome, 00185 Rome, Italy; michele.runci@gmail.com

**Keywords:** synaptic organization, sarcoglycans, cell adhesion, non-muscle organ, tissue homeostasis, epithelial signaling

## Abstract

This scoping review explores the expression patterns and molecular features of sarcoglycans (SGs) in non-muscle organs, challenging the long-standing assumption that their function is confined to skeletal and cardiac muscle. By analyzing evidence from both animal models and human studies, the review highlights the widespread presence of SG subunits in organs, including the nervous system, glands, adipose tissue, oral mucosa, retina, and other structures, with distinct regional and cell-type-specific patterns. Studies on the central nervous system demonstrate a widespread “spot-like” distribution of SG subunits in neurons and glial cells, implicating their involvement in synaptic organization and neurotransmission. Similarly, SGs maintain cellular integrity and homeostasis in glands and adipose tissue. At the same time, the altered expression of SGs is associated with pathological conditions in the gingival epithelium of the oral mucosa. These findings underscore the multifaceted roles of SGs beyond muscle, suggesting that they may contribute to cellular signaling, membrane stability, and neurovascular coupling. However, significant gaps remain regarding SG post-translational modifications and functional implications in non-muscle organs. Future research integrating molecular, cellular, and functional approaches in animal models and human tissues is essential to fully elucidate these roles and explore their potential as therapeutic targets in various diseases.

## 1. Introduction

Sarcoglycans (SGs) are a family of transmembrane glycoproteins essential for the maintenance of muscle cell structure and function [1,2,3]. These proteins, comprising six known subunits (α, β, γ, δ, ε, and ζ), assemble to form the sarcoglycan complex (SGC), a core component of the dystrophin-associated glycoprotein complex (DGC). Together with α- and β-dystroglycans (DGs), and sarcospan (SSPN), SGs establish a molecular bridge between the intracellular cytoskeleton and the extracellular matrix via laminin α2, thereby contributing to sarcolemmal integrity and enabling signal transduction during muscle contraction [4,5,6,7,8,9,10]. Disruption of this system leads to membrane instability and progressive muscle degeneration, as observed in several forms of limb–girdle muscular dystrophies (LGMDs) [11]. Each SG subunit displays tissue-specific expression patterns and distinct structural characteristics. α-, β-, γ-, and δ-SGs are predominantly expressed in skeletal and cardiac muscle, whereas ε-SG (which shares 44% sequence similarity with α-SG) is mainly found in smooth muscle. ζ-SG is structurally related to both γ- and δ-SG. The composition of the SGC adapts to tissue type: α-, β-, γ-, and δ-SGs are typically found in striated muscle, while ε-SG replaces α-SG in smooth muscle [12,13]. Structurally, β-, γ-, and δ-SGs are type II transmembrane proteins with intracellular N-termini, whereas α- and ε-SGs are type I proteins with extracellular N-terminal domains [13]. These differences suggest the existence of at least two distinct sarcoglycan complexes with potentially diverse functions [12,14]. Importantly, the structure and function of SGs are highly dependent on post-translational modifications and molecular interactions. Within the endoplasmic reticulum (ER), SGs undergo N-glycosylation, a sequential process involving the addition of mannose, sialic acid, and N-acetylglucosamine residues, which is essential for protein maturation, membrane targeting, and interaction with DG [3,15,16]. SGs are also localized within lipid-rich membrane microdomains (lipid rafts), composed of cholesterol and sphingolipids, which influence their clustering, localization, and signaling capacity. Moreover, SGs interact with various protein scaffolds, which support their stabilization and functional integration within cellular signaling networks.

While most research has focused on the role of SGs in muscle tissue and related pathologies, there is growing evidence of their expression in non-muscle tissues, including smooth muscle layers of internal organs (gastrointestinal and urogenital tracts, ureters, skin) [17,18,19,20], as well as in the brain, endocrine glands, and adipose tissue. Furthermore, mutations in SG-encoding genes have been linked to non-muscle phenotypes, such as the association between ε-SG mutations and myoclonus–dystonia syndrome, suggesting SG involvement in neuronal signaling and motor control [21,22]. However, the existing literature on SGs in non-muscle tissues primarily focuses on expression patterns, with limited attention to the molecular mechanisms that govern their function, specifically post-translational modifications, lipid interactions, and associations with scaffold proteins. Given that these factors are fundamental to SG structure and activity, it is likely that evaluating SGs in non-muscle contexts without considering these molecular features is incomplete or even misleading. This scoping review aims to explore and synthesize the current knowledge on SGs in non-muscle tissues, focusing primarily on their expression profiles and, secondarily, examining available data on their glycosylation patterns, interactions with lipid membranes, and associations with membrane scaffold proteins. By identifying whether and how these aspects have been investigated outside of muscle, this review seeks to fill a critical gap in the literature and support a more comprehensive understanding of SG functions across different tissue types.

## 2. Materials and Methods

### 2.1. Search Strategy

This scoping review followed the Preferred Reporting Items for Systematic Reviews and Meta-Analyses for Scoping Reviews (PRISMA-ScR) statement [23].

### 2.2. Eligibility Criteria

The primary question was the expression of SG proteins in various healthy non-muscle organs in both human and animal models. The secondary questions were as follows:

• Which SG subunits have been detected in non-muscle organs?
• What experimental or observational methods have been employed to assess SG expression?
• Are there reported associations between SG expression levels and specific pathological or physiological states in non-muscle organs?
• Are there molecular features, such as glycosylation, membrane lipid associations, or scaffold protein interactions, described for SGs in these organs?
• What are the main research gaps identified in the literature?

The eligibility criteria for admission in this scoping review are defined in Table 1.

**Table 1:** Inclusion and exclusion criteria.

Inclusion Criteria	Exclusion Criteria
Clinical and ex vivo human studies	Studies focusing exclusively on muscle
Animal studies	Studies on the expression of SGs in non-muscle organs identified in the muscular coat
Cell-based models	Studies involving sarcoglycanopathies and myoclonic dystonia
Studies that investigate the expression of one or more SG subunits (α, β, γ, δ, ε, and ζ) in non-muscle organs	Case reports, case series, reviews, meta-analyses, letters, editorials, commentaries, communications, supplements, and proceedings papers
Research articles as experimental studies, observational studies, and in vitro/in vivo analyses	Papers without full-text availability
Papers published in English	Papers published in languages other than English

### 2.3. Information Sources

A comprehensive literature search was conducted using the following electronic databases: PubMed/MEDLINE, Scopus, Web of Science, and ScienceDirect. In addition, Google Scholar was consulted to identify relevant grey literature. To ensure comprehensiveness, the search strategy included Boolean operators to identify articles using both MeSH (Medical Subject Headings) terms and free-text keywords: (Sarcoglycan complex OR Sarcoglycans) AND (Non-Muscle Tissue OR Epithelial tissue OR Gland OR Prostate OR Breast OR Liver OR Pancreas OR Spleen OR Nervous system OR Kidney OR Lung OR Connective tissue OR Adipose organ) AND (Immunohistochemistry OR RT-PCR OR Immunofluorescence OR mRNA). The search strategy was adapted for each database to optimize the retrieval of relevant articles (Table 2).

**Table 2:** Database-specific search strategies.

Database	Search Strategy
PubMed/MEDLINE “https://pubmed.ncbi.nlm.nih.gov/ (accessed on 19 February 2025)”	(Sarcoglycan complex OR Sarcoglycans) AND (Non-Muscle Tissue OR Epithelial tissue OR Gland OR Prostate OR Breast OR Liver OR Pancreas OR Spleen OR Nervous system OR Kidney OR Lung OR Connective tissue OR Adipose organ) AND (Immunohistochemistry OR RT-PCR OR Immunofluorescence OR mRNA)
Scopus “https://www.scopus.com/home.uri (accessed on 19 February 2025)”	(“Sarcoglycans” OR sarcoglycan*) AND (“Non-Muscle Tissues”) AND (expression OR “immunohistochemistry” OR “immunofluorescence” OR “RT-PCR” OR mRNA)
Web of Science “https://www.webofscience.com/wos/ (accessed on 19 February 2025)”	(Sarcoglycan complex OR Sarcoglycans) AND (Non-Muscle Tissue OR Epithelial tissue OR Gland OR Prostate OR Breast OR Liver OR Pancreas OR Spleen OR Nervous system OR Kidney OR Lung OR Connective tissue OR Adipose organ) AND (Immunohistochemistry OR RT-PCR OR Immunofluorescence OR mRNA)
ScienceDirect “https://www.sciencedirect.com/ (accessed on 19 February 2025)”	(Sarcoglycan complex OR Sarcoglycans) AND “Non-muscle tissue” AND (Immunohistochemistry OR “RT-PCR” OR Immunofluorescence OR mRNA)
Google Scholar “https://scholar.google.com/ (accessed on 19 February 2025)”	(Sarcoglycan complex OR Sarcoglycans) AND “Non-muscle tissue” AND (Immunohistochemistry OR “RT-PCR” OR Immunofluorescence OR mRNA)

### 2.4. Screening and Selection Process

All articles identified by the search in the various databases were imported into reference management software (Mendeley Desktop, version 1.19.8), where duplicates were removed. After duplication, the titles and abstracts of the remaining studies were independently reviewed by two reviewers (FN and GV) to assess their relevance to the research questions. Studies containing the relevant search terms in the title and/or abstract were selected for full-text review. The two reviewers (FN and GV) independently reviewed the full-text articles of potentially relevant studies based on predefined eligibility criteria. Studies that met all criteria were included for data extraction, while those that did not meet the criteria were excluded, and the reasons for exclusion were recorded. Any disagreements between the two reviewers at this stage were discussed and resolved by consensus. If disagreement persisted, a third independent reviewer (JF) was consulted.

### 2.5. Data Extraction

The two reviewers (FN and GV) independently extracted the following data from the selected articles: authors’ names, year of publication, type of study, tissue/organ types examined, SG subunits studied, and species/models. In cases where discrepancies emerged between data extractions, the third reviewer (JF) was consulted to resolve any disagreements.

### 2.6. Data Synthesis

The extracted data from the included studies were synthesized narratively and organized by organ type. Subunits were mapped based on reported mRNA and protein expression across tissues, and evidence from human and animal studies was clearly distinguished. Consistencies and discrepancies among studies were noted, and results were summarized in descriptive tables and figures.

## 3. Results

### 3.1. Study Selection

**Figure 1:**
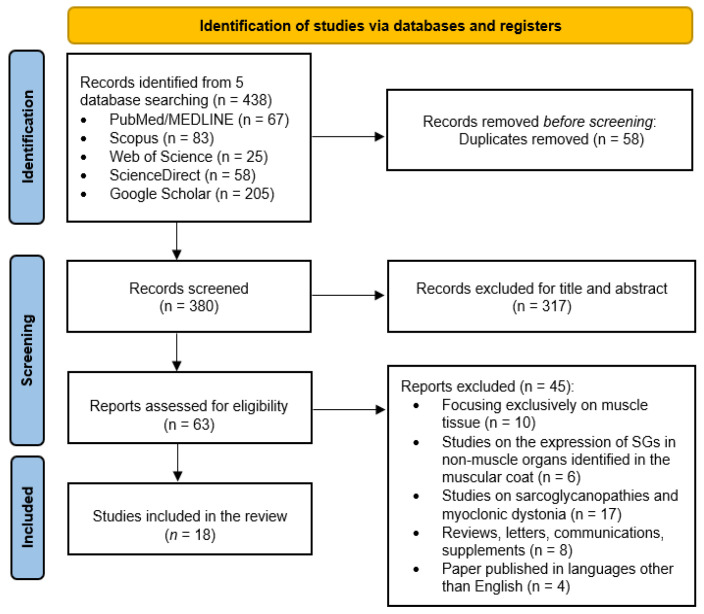
PRISMA flowchart summarizing the article selection process.

The search process identified 438 articles. After removing duplicates, 380 articles remained for initial screening. From these, 317 papers were dismissed based on their titles and abstracts, primarily for being unrelated to SGs involvement in non-muscle organs. A comprehensive review of the remaining 63 articles revealed that 18 studies met the inclusion criteria and were included in the final analysis. Figure 1 shows the workflow of the selected studies.

### 3.2. Characteristics of the Included Studies

The included studies were published between 2000 and 2022, and employed various experimental approaches to investigate SG expression. Immunohistochemistry and immunofluorescence [16,24,25,26,27,28,29,30,31,32,33,34,35,36,37], fluorescence in situ hybridization (FISH) [16], and in situ hybridization (ISH) [38] were widely used to examine protein expression and localization in tissue samples. For immunohistochemistry, sections were examined using a laser scanning confocal microscope [16,24,25,26,27,28,29,30,31,32,33,34,35,36,37] or a fluorescence microscope [16,25]. For FISH, fluorophore-labeled probes were observed by a confocal microscope [16], whereas ISH employed radiolabeled cRNA probes [38]. Real-time reverse transcriptase–polymerase chain reaction (RT-PCR) [25,26,27,28,30,34,36,38,39,40], Western blot [25,26,28,30,33,39,40], and Northern Blot [38] were frequently applied to analyze mRNA and protein expression levels.

**Figure 2:**
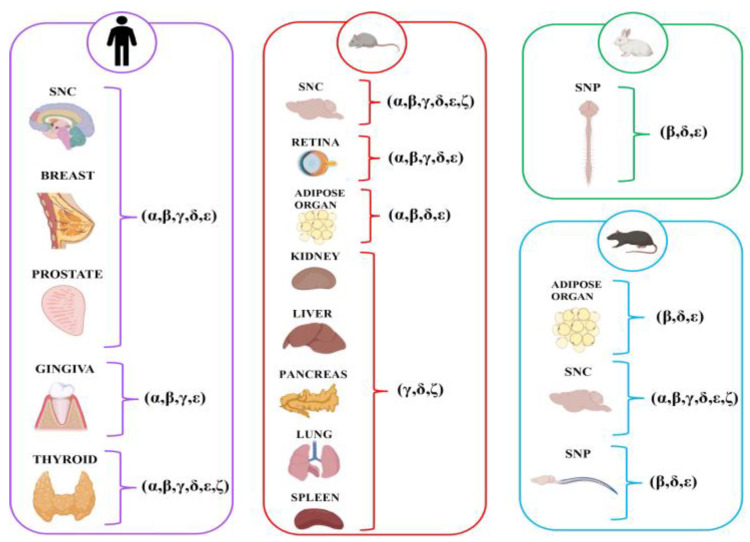
Expressions of SGs in non-muscle organs categorized by human and animal models (mouse, rat, and rabbit).

Studies have examined a wide range of non-muscle organ types. The nervous system, divided into central [16,24,26,28,30,31,37,38] and peripheral [25,33], and adipose [39,40] have been studied. Other organs/tissues explored included the glands (as breast [27], prostate [36], thyroid [29], pancreas [30]), and oral gingival mucosa [32,35]. The most investigated SG subunit was represented by ε [16,24,25,26,27,28,29,31,32,33,34,35,36,37,38,39,40], followed by β [24,25,26,27,28,29,31,32,33,34,36,37,39,40] and δ [24,25,26,27,28,29,30,31,34,36,37,40], γ [24,26,27,28,29,30,31,32,34,35,36,37], α [24,26,27,28,29,31,34,35,36,37,40], and ζ [25,26,29,30,31,37]. Regarding the species, the research included animal models and human tissue samples [27,28,29,32,35,36]. Rats [24,25,31,37,38,39] and mice [16,26,34,40] were frequently used. Only one study used the rabbit species [32]. Furthermore, several studies investigated human disease models, exploring conditions such as benign prostatic hyperplasia and prostate adenocarcinoma [36], fibrocystic mastopathy and breast fibroadenoma [27], Hashimoto’s thyroiditis [29], and bisphosphonate-related osteonecrosis of the jaw [32,35]. The findings related to the expression of SGs, as reported in the studies, are presented in Figure 2.

### 3.3. Overview of SG Expression Across Non-Muscle Organs

Current evidence highlights both widespread and tissue-specific expression patterns of SG subunits across organ systems. Among them, β- and δ-SGs are the most consistently detected, being expressed in all major tissues analyzed, including the CNS [24,26,28,31,34,37], PNS [25,33], glands [27,29,36], and adipose tissue [39,40]. Their broad presence suggests a constitutive role across both neuronal and non-neuronal compartments. ε-SG also shows widespread expression but with a more variable distribution. It is abundantly expressed in the CNS [16,24,26,28,31,37,38], including in the Müller glial and ganglion cells of the retina [34], in Schwann cells of the PNS [25,33], in the prostate, breast, and thyroid [27,29,36], and in the gingival epithelium [32,35]. However, this subunit is detectable in certain organs, such as the liver, kidney, spleen, and testis, while in adipose tissue, it is only detectable during the adipogenic process [40], indicating potential tissue-specific regulation. ζ-SG displays a more restricted pattern, with predominant expression in the brain [24,30,37] and limited or absent mRNA signals in other tissues such as the pancreas, kidney, or liver [30]. By contrast, γ-SG is primarily enriched in the muscle and lung [30], with only low expression detected in the CNS (mainly in the cerebellum and brainstem) [30,31,37], and it is absent from Schwann cells and adipose tissue [25,40].

**Figure 3:**
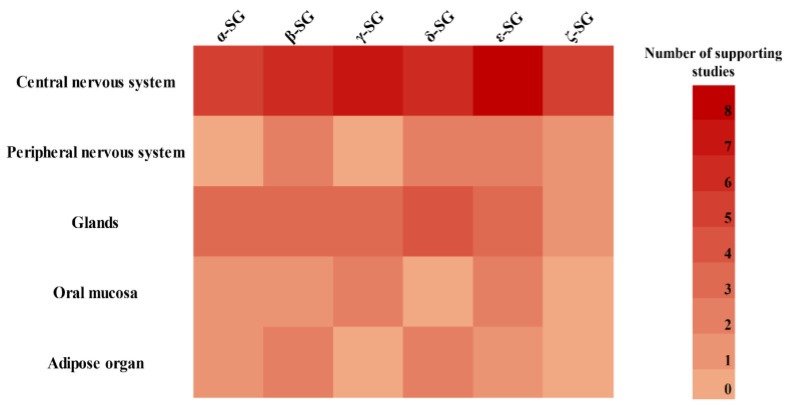
Heatmap summarizing the expression of SG subunits across non-muscle organs. Color intensity reflects the frequency of reported expression in the literature based on the number of studies identifying each SG subunit in a given organ.

Other subunits (α- and γ-SGs) also exhibit more tissue-restricted profiles. α-SG is detected in cerebral cortex neurons and astrocytes [24,28,31,37], in glands [27,29,36], the oral epithelia [35], and adipose organ [40], but is consistently absent from the retina [34] and PNS [25,33]. γ-SG was primarily found in the brainstem and cerebellar regions [24,31,37], and in epithelial and oral mucosal tissues [27,29,32,35,36], but was absent in peripheral nerves and adipose tissue [25,33,39,40]. To better visualize these distribution patterns, a heatmap was generated to summarize the expression of individual SG subunits based on evidence from the included studies (Figure 3).

### 3.4. Central Nervous System (CNS)

#### 3.4.1. Animal Studies

Xiao and LeDoux [38] detailed the molecular characteristics and expression patterns of the rat ε-SG gene. First, it was shown that this gene encodes a 437-amino-acid protein highly conserved in rats, mice, and humans, especially in its 46-amino-acid N-terminal signal sequence, which directs the protein to the cell membrane. While the rat and mouse sequences are identical, subtle variations were found in the human sequence that could affect species-specific aspects of SG function. In addition, the structural analysis showed that the extensive extracellular domain contains a conserved asparagine for glycosylation and four cysteine residues. Northern blot analysis of ε-SG mRNA expression showed moderate levels in the brain. Furthermore, quantitative RT-PCR confirmed the presence of ε-SG mRNA, revealing minimal developmental variation in the hippocampus, unlike the significant decrease in muscle tissue, where neonatal levels were over ten times higher than in adults. The in situ hybridization of brain sections demonstrated a widespread and uniform distribution of ε-SG mRNA in neurons, with the strongest signals found in areas with high neuronal density, such as the pyramidal cell layer of the hippocampus, cerebellum, and cerebral cortex, as well as in various brainstem, midbrain, thalamic, and hypothalamic nuclei. Clear hybridization signals observed in fiber pathways and white matter tracts (cerebellar peduncles and corpus callosum) suggested that glial cells also express ε-SG mRNA.

Complementary findings by Chan et al. [16] further extended these insights by revealing that ε-SG mRNA and protein have a widespread presence across the mouse brain, as demonstrated through FISH and immunohistochemistry. High levels of ε-SG mRNA expression were particularly evident in the olfactory bulb’s mitral cell layer and the cerebellum’s Purkinje cell layer. Moreover, significant expression was detected in various monoaminergic cell clusters, including dopaminergic neurons within the substantia nigra pars compacta (SNc), substantia nigra pars reticulata (SNr), and ventral tegmental area (VTA), as well as serotonergic neurons in the dorsal raphe nucleus and noradrenergic neurons in the locus coeruleus. In contrast, moderate expression levels were observed in the hippocampus, multiple hypothalamic nuclei, and the amygdala, whereas lower levels were noted in regions such as the neocortex, globus pallidus, and most thalamic nuclei.

Shiga et al. [30] investigated the tissue-specific expression of ζ-SG, reporting its predominant expression in the brain. In contrast, γ-SG exhibited lower levels of expression. A more detailed regional analysis revealed a uniform distribution of ζ-SG throughout the brain, whereas γ-SG was abundant in specific areas such as the cerebellum and pons/medulla. Post-translational analysis revealed that ζ-SG, like other SGs and DGs, undergoes N-linked glycosylation, as evidenced by a shift in molecular weight after PNGase F treatment. Deglycosylation resulted in a reduction of ζ-SG’s molecular weight, while maintaining a doublet migration pattern. To better understand the role of ζ-SG in SGC formation, CHO cells were used to perform the co-expression of all six known SGs with DG, which is crucial for the transportation of the SG complex to the cell plasma membrane. Similar molecular weight shifts as described above were observed for all SG and DG proteins expressed in CHO cells following PNGase F treatment. Concerning membrane interactions and scaffold proteins, ζ-SG was detected in both the plasma membrane and intracellular compartments, including the ER and Golgi apparatus. Cell surface biotinylation and immunoprecipitation experiments in CHO cells demonstrated that ζ-SG forms stable complexes with α-, β-, δ-, and ε-SG, but not with γ-SG. In particular, the simultaneous expression of all six known SGs with DG in CHO cells led to the identification of four distinct SG complexes at the membrane: (1) α- β- γ- δ, (2) α- β- ζ- δ, (3) ε- β- γ- δ, and (4) ε- β- ζ- δ. These findings indicate that ζ-SG can functionally substitute for γ-SG in SGC assembly and integrate into both α- and ε-based complexes. Moreover, SG complexes were also found to associate with DG, regardless of whether ζ-SG is present or not, suggesting that ζ-SG-containing complexes can exist as a subcomplex of the entire DGC in vivo.

Immunofluorescence studies in the cerebral and cerebellar cortices confirmed the presence of multiple SG subunits [24,37]. The research by Vermiglio et al. [24] reported that all tested SGs (α, β, γ, δ, and ε) are expressed in the rat cerebral cortex, where they exhibit a “spot-like” fluorescence pattern with spots (0.5–2 μm in diameter) around neuronal somas. All SG expressions were marked in the cerebellar cortex in the Purkinje cell layer, while the granular and molecular layers showed low or absent staining. Regarding localization, SG expression was also observed in glial cells, mainly in the central portion rather than around the soma.

Cutroneo et al. [37] further corroborated these findings by showing that all SGs (α, β, γ, δ, ε, and ζ) are expressed in rat neurons across the hippocampus, cerebral cortex, and cerebellar cortex. In these regions, the proteins exhibited a spot-like fluorescence pattern (0.5–2 μm in diameter) predominantly localized in the soma of pyramidal and granular neurons. Specifically, in the hippocampus, SGs were more expressed in pyramidal neurons of some Cornu Ammonis regions (CA1, CA2, and CA3) and dentate gyrus granular cells, which displayed more minor fluorescence spots than pyramidal neurons. In the cerebellar cortex, SG expression was evident in all layers, with a high spot-like immunostaining predominantly localized to the Purkinje cell layer. Additionally, the colocalization of each SG with the GABA_A receptor indicated colocalization at the soma level, confirming the neuronal expression of SGs.

Boulay et al. [26] investigated the expression of the SG complex in the cerebrovascular system and its regulation by astrocytic Connexin 30 (Cx] 30). RT-PCR analysis performed on purified brain vessels demonstrated the presence of transcripts encoding all known SG subunits (α, β, δ, ε, γ, and ζ) as well as SSPN, indicating that the full SG complex is transcriptionally active in the cerebrovascular system. Among these subunits, δ- and ε-SG were particularly expressed in larger-caliber vessels (diameter > 100 μm). To explore the regulatory influence of astrocytic Cx30 on vascular SG expression, the authors examined Cx30-knockout mice. Transcriptomic analysis revealed that most SG subunits, including α- and β-SG, maintained comparable expression levels between wild-type and knockout animals. However, the expression of Sgcg, the gene encoding γ-SG, was significantly upregulated in the absence of Cx30. Western blot analysis confirmed that this transcriptional upregulation of γ-SG translated into increased protein levels in brain vessels.

Rizzo et al. [31] investigated the expression of SGs in the cerebral and cerebellar cortices of rats. In the cerebral cortex, single immunofluorescence reactions revealed that all six SG subunits are expressed in a distinctive “spot-like” pattern, with spots (0.5–2 μm in diameter) primarily localized around the soma of neurons and glial cells. The statistical analysis of spot counts across different cortical regions indicated significant variability in subunit expression, particularly for α-, β-, and γ-SG, between anterior and posterior cortical areas. Conversely, in the cerebellar cortex, SG expression was consistently uniform across different regions, with the same staining pattern observed around the soma of Purkinje cells and glial cells.

In wild-type mouse retinae observed by Fort et al. [34], SGs and SSPN exhibited a distinct expression profile compared to skeletal muscle, with their localization and abundance appearing largely independent of dystrophin. Using RT-PCR and Western blot analyses in both wild-type and *mdx3cv* mice, the authors confirmed the presence of β-, δ-, γ-, and ε-SG subunits, along with SSPN, at both mRNA and protein levels. Among these, ε-SG and β-SG were the most abundantly expressed, while α-SG was undetectable in the retina of either genotype. Notably, even in *mdx3cv* mice, characterized by a severe reduction in Dp71 (a short dystrophin subunit), the expression levels and spatial distribution of SGs and SSPN remained unchanged. Immunofluorescence analyses revealed that SGs and SSPN localize predominantly at the inner limiting membrane (ILM), corresponding to the end feet of Müller glial cells (MGCs), and at the outer limiting membrane (OLM), with additional expression in the ganglion cell layer (GCL) and inner nuclear layer (INL). Double immunolabeling with markers for MGC (glutamine synthetase) and ganglion cell axons (NF68) confirmed that SGs and SSPN localize to both glial and neuronal compartments, with strong signal overlap observed particularly for ε-SG. Importantly, SGs did not colocalize with dystrophin in photoreceptor terminals or dendritic processes, and their localization was not disrupted in dystrophin-deficient mice. Based on these findings, the authors proposed the presence of two distinct SG–SSPN assemblies: one associated with Dp71 at the MGC end feet and another located in the outer retina, potentially interacting with as yet unidentified molecular partners.

#### 3.4.2. Human Studies

Anastasi et al. [28] investigated the expression and localization of SGs in the human cerebral cortex. The study found that all SG subunits (α-, β-, γ-, δ-, ε-, and ζ-SG) are present in large neurons of the cerebral premotor cortex, with immunostaining localized predominantly along the cell surface. Three-dimensional reconstructions of full-thickness sections verified that SG immunoreactivity is distributed throughout the neuronal membranes. Furthermore, double labeling with neuronal and glial markers demonstrated that SGs are expressed in neuronal and astrocytic populations, with colocalization patterns indicating potential functional interactions at the cell surface.

Table 3 summarizes the available studies investigating SG expression in the CNS across both animal and human models.

**Table 3:** Overview of the studies included for the CNS.

Study (Authors, Year)	Study Design	Tissue/Organ/Cell Types Examined	Types of SG Proteins Studied	Species/Models
Xiao and LeDoux (2003) [38]	Molecular study (Northern analysis, RT-PCR and ISH)	Neural (cerebellar cortex, striatum, cerebral cortex, thalamus, hippocampus)	ε	Rat [16]
Vermiglio et al. (2011) [24]	Immunofluorescence study	Cerebral and cerebellar cortex (neurons and glial cells)	α, β, γ, δ, ε	
Cutroneo et al. (2015) [37]	Immunohistochemical study	hippocampus, cerebral and cerebellar cortex	α, β, γ, δ, ε, ζ	
Rizzo et al. (2018) [31]	Immunofluorescence study	Cerebral cortex (neurons and glial cells) Cerebellar cortex (neurons and glial cells)	α, β, γ, δ, ε, ζ	
Chan et al. (2005) [16]	Immunohistochemistry and molecular (FISH) study	Olfactory bulb (mitral cell layer) Cerebellum (Purkinje cell) Hippocampal formation and neocortex Monoaminergic cell groups and brainstem nuclei	ε	Mouse
Shiga et al. (2006) [30]	Immunofluorescence and molecular (RT-PCR and immunoprecipitation) study	Brain	γ, ζ	
Fort et al. (2005) [34]	Immunohistochemistry and molecular (RT-PCR) study	Retina (Müller and ganglion cells)	α, β, γ, δ, ε	
Boulay et al. (2015) [26]	Immunofluorescence and molecular (RT-PCR, Western blot) study	Cerebrovascular system (brain vessels, cortex, and hippocampus)	α, β, γ, δ, ε, ζ	
Anastasi et al. (2012) [28]	Immunohistochemical and molecular study (RT-PCR, Western Blot)	Cerebral cortex (neurons and astrocytes)	α, β, γ, δ, ε, ζ	Human

### 3.5. Peripheral Nervous System (PNS)

#### 3.5.1. Animal Studies

In the PNS, SGs are prominently expressed in the outer regions of nerve fibers, as described by Imamura et al. [33]. Western blot analysis of various mouse tissue lysates showed that the 46 kDa ε-SG is widely expressed, with the highest levels observed in peripheral nerve tissue. Immunofluorescence analysis of rabbit peripheral nerve cryosections revealed that β-SG, δ-SG, and ε-SG, together with α-DG and β-DG, are localized along the outer region of nerve fibers. In contrast, α-SG, γ-SG, and SSPN were not detected in these regions. Double staining with antibodies against ε-SG and neurofilament or laminin B1 chain indicated that ε-SG is not located on the axon but near the basal lamina in close association with Schwann cells. In addition, ε-SG colocalized with both β- and δ-SGs at the outer region of the myelin sheath and showed colocalization with DGs, Dp116, and utrophin. Wheat germ agglutinin (WGA) affinity chromatography of peripheral nerve lysates confirmed these findings. In the WGA-bound fraction from peripheral nerve, immunoblotting detected β-SG, δ-SG, and ε-SG along with DGs, Dp116, and utrophin, whereas α-SG, γ-SG, and SSPN were absent even at high detection sensitivities. Immunoprecipitation of this fraction with an anti-ε-SG antibody further confirmed that β-, δ-, and ε-SGs assemble into a complex with DGs and Dp116.

Cai et al. [25] provided complementary evidence for the expression of SGs in Schwann cells of the PNS, where they contribute to the structural integrity of the myelin sheath. Through RT-PCR and Western blot analyses, the authors demonstrated that β-, δ-, ε-, and ζ-SGs are expressed in adult rat sciatic nerves and cultured Schwann cells, even in the absence of neuronal input. In contrast, α- and γ-SGs were not detected. Immunofluorescence localized these subunits to the abaxonal membrane of Schwann cells near the basal lamina. Notably, SG expression occurred before the onset of myelination and was upregulated by the presence of neurons, independent of myelin assembly. Co-immunoprecipitation experiments showed that β-, δ-, ε-, and ζ-SGs form a stable heteromeric complex with Dp116 and α/β-DGs. In BIO14.6 hamsters, which lack δ-SG due to a genetic deletion, the entire complex was destabilized, with reduced levels of Dp116 and α-DG in membrane fractions. Additionally, α-DG was also reduced in the supernatant. In contrast, β-DG, utrophin, and syntrophin levels remained unchanged. Electrophoretic analysis showed altered mobility of α-DG between muscle and nerve tissues. Structural analysis revealed abnormalities in the myelin sheath, including excessive folding, internal splitting, and occasional axonal compression, as well as disruption of Schmidt–Lanterman incisures. Despite normal levels of myelin proteins (MAG, MBP, P0), these structural defects were associated with mild conduction delays under thermal stress at temperatures below 20 °C.

#### 3.5.2. Human Studies

To date, there is a paucity of human data on SG expression in the PNS.

Table 4 summarizes the available studies investigating SG expression in the PNS across both animal and human models.

**Table 4:** Overview of the studies included for the PNS.

Study (Authors, Year)	Study Design	Tissue/Organ/Cell Types Examined	Types of SG Proteins Studied	Species/Models
Cai et al. (2007) [25]	Immunofluorescence and molecular study (RT-PCR and Western blot)	Peripheral nervous system (sciatic nerves and Schwann cell cultures)	α, β, γ, δ, ε, ζ	Rat Hamster
Imamura (2000) [33]	Immunofluorescence and molecular (immunoprecipitation and Western blot) study	Peripheral nerves (sciatic, femoral and tibial)	α, β, γ, δ, ε	Rabbit

### 3.6. Glands

#### 3.6.1. Animal Studies

Additional insights into the composition and tissue distribution of SG complexes were provided by Shiga et al. [30]. When examining mouse tissues by RT-PCR, ζ-SG mRNA levels were highest in the brain and very weak in other tissues, including those of glandular origin (e.g., pancreas). In contrast, δ-SG exhibited broad and abundant expression across all tissues examined.

#### 3.6.2. Human Studies

Arco et al. [27] analyzed the distribution of SGs in breast glandular epithelial cells. In normal glandular breast tissue, single immunofluorescence analysis revealed a uniform staining pattern of all tested SG subunits (α-, β-, γ-, δ-, ε-SGs) in polyhedral epithelial cells. Serial sectioning combined with three-dimensional reconstruction confirmed that SG staining spanned the entire cellular thickness, with expression evident in both epithelial and myoepithelial cells, as validated by double localization with α-SMA and DAPI. In contrast, breast tissue affected by fibrocystic mastopathy and fibroadenoma exhibited a marked reduction in SG expression. In these pathological specimens, immunofluorescence showed only isolated, faint fluorescent spots, and RT-PCR analysis demonstrated significantly decreased mRNA levels of all SGs compared with normal tissue.

Cutroneo et al. [36] extended the investigation to the prostatic gland. In normal prostate, single immunofluorescence reactions demonstrated clear expression of α-, β-, γ-, δ-, ε-SGs, with a well-defined distribution localized to the apical, lateral, and basal areas of the epithelial cells arranged in a single layer. Double-localization with α-SMA and DAPI confirmed that SGs are present in epithelial and myoepithelial cells. However, these patterns were altered in prostatic diseases. Benign prostatic hyperplasia displayed only a limited SG staining pattern confined to the apical areas of epithelial cells. In contrast, in prostatic adenocarcinoma, SG expression was absent in both epithelial and myoepithelial cells. RT-PCR analysis further confirmed decreased SG transcript levels in these pathological conditions.

Favaloro et al. [29] performed an immunofluorescence study on thyroid tissue in both healthy and pathological conditions to evaluate the expression of SGs (α, β, γ, δ, ε, and ζ) and αvβ3-integrin. In healthy subjects, single-localization immunofluorescence in thyrocytes showed a uniform staining pattern for all six SG subunits and αvβ3-integrin. In contrast, thyrocytes from subjects with Hashimoto’s thyroiditis exhibited a decreased staining pattern for all SGs and αvβ3-integrin, while merged images demonstrated an absence of SG signal in some of these cells. Quantitative pixel intensity analysis corroborated these observations, revealing significantly lower protein expression levels in pathological samples compared to controls.

Table 5 summarizes the available studies investigating SG expression in the glands across both animal and human models.

### 3.7. Oral Mucosa

#### 3.7.1. Animal Studies

There is little to no animal data on SG expression in the oral mucosa for the studies reviewed.

#### 3.7.2. Human Studies

Nastro-Siniscalchi et al. [32] investigated the effects of bisphosphonate treatment on oral mucosa, explicitly focusing on the expression of proteins critical for cell viability and signaling, including SGs. In control samples, the immunofluorescence analysis showed a clear and consistent expression of three SG subunits (β, γ, and ε) within the basal lamina. In samples from bisphosphonate-treated subjects without osteonecrosis, the expression of these SGs was markedly reduced. Basal lamina exhibited an almost complete absence of detectable staining for these SGs, which indicated a disruption of normal protein expression patterns. Conversely, in bisphosphonate-treated samples from patients with osteonecrosis, increased SG staining was observed on the basal lamina, with a concomitant qualitative and quantitative rise in vascular-associated fluorescence.

Further supporting these observations, the study by De Ponte et al. [35] on oral mucosa biopsies from untreated subjects revealed a regular and continuous expression of α-, ε-, and γ-SGs from the basal keratinocyte layer to the superficial layers. In biopsies from patients treated with zoledronate for 24 months, a general decrease in SG fluorescence pattern was noted, while in specimens from patients undergoing bisphosphonate therapy for 36 months, SG expression was nearly undetectable. Quantitative analysis using fluorescence intensity profiles confirmed these findings, with control samples displaying distinct peaks of SG fluorescence that were markedly diminished or absent in long-term bisphosphonate-treated specimens.

Table 6 summarizes the available studies investigating SG expression in the oral mucosa across both animal and human models.

### 3.8. Adipose

#### 3.8.1. Animal Studies

Groh et al. [40] investigated the expression and functional importance of SGs in white adipose tissue. RT-PCR and protein analyses in freshly isolated white adipocytes demonstrated the presence of α-, β-, and δ-SGs, along with SSPN and DG. In contrast, γ- and ε-SG are not expressed in these cells. Immunofluorescence confirmed membrane localization of these SGs in adipocytes, and expression levels were consistent with transcript abundance. Functional experiments using mouse models deficient in individual DGC components revealed that the adipocyte SG complex functions as a tightly interdependent unit: loss of either β- or δ-SG led to destabilization of the entire complex, including SSPN. This also resulted in a significant reduction in the glycosylated form of α-DG, thereby compromising its ability to bind laminin despite the continued presence of the core α-DG protein. In contrast, the deletion of α-SG did not affect the expression of the adipocyte SG complex.

Complementing these observations, Romo-Yáñez et al. [39] investigated the expression and regulation of SGs and associated DGC components in white adipose tissue, with a focus on adipocyte differentiation. Their study demonstrated that β-, δ-, and ε-SGs are consistently expressed at the mRNA level throughout the adipogenic process in both visceral and subcutaneous fat depots of adult rats. These transcripts were detected by RT-PCR and remained present throughout all stages of adipocyte differentiation in vitro, including preconfluent preadipocytes, quiescent preadipocytes, committed adipocytes, and mature adipocytes. In contrast, no expression of α- or γ-SG was reported. Additionally, SGs were co-expressed with key DGC components, including β-dystroglycan and utrophin. Both proteins were present at all differentiation stages, implying that a functional DAPC is assembled in adipose cells. In addition, multiple subunits of syntrophins (α, βI, βII, and cII) and dystrobrevins (α and β) were expressed, forming a cytoplasmic interface for signaling and cytoskeletal anchorage. Among these, α-dystrobrevin was selectively expressed only after adipogenic commitment. Conversely, βI-syntrophin displayed depot-specific regulation, with stable expression during visceral adipogenesis and a progressive decrease in subcutaneous adipocytes.

#### 3.8.2. Human Studies

Currently, direct studies on SG expression in human adipose tissue are limited.

Table 7 summarizes the available studies investigating SG expression in the adipose across both animal and human models.

### 3.9. Other Organs

#### 3.9.1. Animal Studies

Xiao and LeDoux. [38] investigated the expression of ε-SG mRNA in non-neural tissues. Using Northern analysis, ε-SG transcript was present in all rat tissues examined, with the highest levels detected in the kidney, moderate in the lung, and lower levels in the liver, spleen, and testis. Shiga et al. [30] also focused on ζ-SG expression in other organs. The RT-PCR analysis of mouse tissues revealed that ζ-SG mRNA levels are faintly detectable in the liver, lung, and spleen. In contrast, γ-SG exhibited high expression in organs such as the lung, while δ-SG showing broad expression in all examined tissues.

#### 3.9.2. Human Studies

There is limited direct evidence from human studies.

Table 8 summarizes the available studies investigating SG expression in the other organs across both animal and human models.

## 4. Discussion

This scoping review highlights the widespread and heterogeneous expression of SGs across various non-muscle organs, including the central and peripheral nervous systems, glands, oral mucosa, and adipose tissue. Traditionally studied in the context of muscle, SGs are increasingly recognized as structural and possibly signaling components in multiple cellular environments. The data reviewed here reveal not only the presence of SGs in diverse organ types but also substantial variability in subunit composition, regional distribution, and cellular localization, features that suggest functional specialization beyond their canonical role in the DGC.

### 4.1. Expression and Functional Roles of SGs in Non-Muscle Organs

#### 4.1.1. CNS

The expression of SGs within the CNS is both widespread and subunit-specific, suggesting distinct and possibly specialized roles beyond their classical functions in muscle. Multiple studies have consistently reported strong expression of ε- and ζ-SG in neurons and glial cells across various brain regions, including the cortex, hippocampus, cerebellum, thalamus, and monoaminergic nuclei [16,30,37,38]. In particular, ζ-SG appears to be predominantly brain-specific, suggesting possible neural specificity [30]. Immunohistochemical studies reveal that SGs are not only widely expressed but also follow a distinct “spot-like” distribution around neuronal somas, particularly in pyramidal and granular neurons, as well as in glial cells [24,31,37]. This spot-like pattern, uniform in some regions (e.g., cerebellum) and variable in others (e.g., cortex), could represent membrane microdomains implicated in synaptic stability or signal transduction. Despite these morphological observations, the exact molecular composition and function of these SG-positive spots remain unknown. In humans, Anastasi et al. [28] confirmed the presence of all six SG subunits in the cerebral premotor cortex, expressed in both neurons and astrocytes. This evidence not only validates the rodent findings but also suggests that SGs could play conserved and functionally relevant roles in the human brain. The membrane-associated distribution observed in full-thickness reconstructions reinforces the idea that SGs may contribute to membrane specialization or intercellular signaling at neuron–glia interfaces.

An interesting case is presented by Shiga et al. [30], who examined both the tissue distribution and biochemical assembly of ζ-SG. While ζ-SG showed strong expression in the brain, it was weakly detectable in skeletal muscle and peripheral tissues. In contrast, γ-SG displayed the opposite expression pattern, suggesting that these two subunits are deployed in a mutually exclusive, tissue-specific manner. In addition, in CHO cell models, ζ-SG was able to assemble into stable DGC complexes when co-expressed with other SG subunits and DG. These complexes mimicked those containing γ-SG, but ζ- and γ-SG did not co-assemble, implying a level of structural redundancy as well as functional exclusivity. These results seem to reflect a divergence between biochemical competence and physiological deployment. The ability of ζ-SG to form complexes in vitro does not imply its incorporation into muscle DGCs in vivo, likely due to transcriptional or trafficking constraints. Instead, ζ-SG could fulfill unique roles in neural-specific DGC variants, particularly in regions where γ-SG is absent. These findings underscore the modularity of the SG complex and highlights the need for in vivo validation to determine whether ζ-SG–based complexes support distinct signaling or structural roles in the CNS.

A key functional insight comes from the observed co-localization of SGs with GABA_A receptors, particularly in hippocampal pyramidal neurons and cerebellar Purkinje cells [37]. This spatial association suggests a critical role for SGs, particularly ε-SG, as scaffolding or regulatory elements in inhibitory synaptic domains. Supporting this hypothesis, the study by Cazurro-Gutiérrez et al. [41] provides compelling evidence that ε-SG directly contributes to the stabilization of GABAergic synapses, influencing receptor clustering and tonic inhibitory transmission. In ε-SG-deficient mice, these functions are impaired, leading to altered GABAergic neurotransmission, disrupted synaptic inhibition, and motor disturbances resembling myoclonus-dystonia (MD), a movement disorder associated with SGCE (ε-SG gene) mutations. The evidence suggests that SGCE plays a crucial role in synaptic function, and its dysfunction in MD may contribute to an imbalance in neurotransmission, particularly a deficit in GABAergic inhibitory signaling.

The CNS-centered view of SGs contrasts with their expression and likely function in the retina. Fort et al. [29] demonstrated that in both wild-type and Dp71-deficient mouse retinas, multiple SG subunits, notably β-, δ-, γ-, and ε-SG, are expressed at the inner and outer limiting membranes, where they predominantly colocalize with Müller glial cell end feet. This distribution occurs independently of dystrophin, indicating that SGs could participate in alternative membrane complexes in the retina, distinct from canonical DGC assemblies. This distinctive pattern suggests that retinal SGs could support non-synaptic functions, such as maintaining glial polarity, epithelial adhesion, or barrier function, rather than participating directly in neurotransmission. Their persistent expression in dystrophin-deficient conditions further implies the existence of alternative membrane complexes or molecular partners yet to be identified. Therefore, the divergence between the brain and retina underscores the context-dependent versatility of SGs. Unlike in the brain, where ε-SG loss affects GABAergic synapses and contributes to motor dysfunction associated with MD, the functional implications of retinal SGs remain speculative but could involve non-synaptic scaffolding roles.

Adding further complexity, Boulay et al. [26] conducted one of the few focused investigations into the expression of SGs in the cerebrovascular system, revealing important but still largely unexplored aspects. The study identified all SG transcripts, as well as SSPN, in purified mouse brain vessels. Notably, δ- and ε-SG were enriched in larger vessels, suggesting spatially regulated expression. These findings raise the possibility that SGs contribute to the molecular architecture of the neurovascular unit, perhaps supporting blood–brain barrier integrity or mechanotransduction. However, no cellular localization or functional data were provided to distinguish expression in endothelial or astroglial components. Of particular interest is the upregulation of γ-SG in Cx30-deficient mice, suggesting that astrocyte-endothelial communication mediated by gap junctions could influence the molecular composition of the vascular DGC. Nevertheless, the biological relevance of this upregulation remains unclear in the absence of mechanistic data.

#### 4.1.2. PNS

Compared to the CNS, the expression and function of SGs in the PNS remain less explored but nonetheless reveal important tissue-specific features. The available data indicate that SGs are expressed in peripheral nerves with a distinct subunit composition and spatial localization, suggesting a specialized role in Schwann cell function and myelin architecture [25,33]. Immunohistochemical and biochemical studies show that β-, δ-, and ε-SG are the predominant subunits present in peripheral nerve fibers, where they localize to the outer region of the myelin sheath, particularly the abaxonal membrane of Schwann cells. In contrast, α-, γ-SG, and SSPN appear to be absent or below detection thresholds in this compartment [33]. These SGs co-localize with known DGC components such as α- and β-DG, Dp116, and utrophin, suggesting the existence of a Schwann cell-specific variant of the DGC. Importantly, the functional role of SGs in the PNS extends beyond structural presence. Co-immunoprecipitation studies in tissues and cultured Schwann cells demonstrate that β-, δ-, ε-, and ζ-SG form stable complexes with Dp116 and DGs even in the absence of neuronal input [25]. The formation of these complexes appears to be developmentally regulated and responsive to axonal signals, as SG expression increases in the presence of neurons even before active myelination occurs. This suggests that SGs may be involved in Schwann cell differentiation or in preparing the membrane environment for myelin formation.

Evidence from δ-SG–deficient BIO14.6 hamsters further supports a functional role for SGs in myelin integrity. In these animals, the absence of δ-SG leads to destabilization of the DGC complex, reductions in Dp116 and α-DG levels, and ultrastructural abnormalities of the myelin sheath, including excessive folding and disrupted Schmidt–Lanterman incisures [25]. These defects occur despite preserved expression of major myelin proteins such as MBP, MAG, and P0, indicating that SGs contribute to myelin organization independently of canonical myelin gene expression. While functional deficits were subtle under normal conditions, thermal stress exacerbated nerve conduction delays, implying that SG-containing complexes may confer mechanical or homeostatic resilience to the peripheral myelin sheath.

These observations suggest that, in contrast to their synaptic roles in the CNS, SGs in the PNS may function primarily as scaffolds for cytoskeletal or ECM interactions that stabilize Schwann cell morphology or regulate myelin architecture under stress. The absence of α- and γ-SG, which are essential in muscle tissue, also reinforces the idea of tissue-specific DGC configurations tailored to the mechanical and molecular environment of peripheral nerves. Notably, human data on SG expression in the PNS remain lacking, leaving open questions about the translational relevance of these findings. Future investigations should focus on the dynamics of SG complex assembly during development and regeneration, as well as its interaction with axonal cues and potential role in human peripheral neuropathies.

#### 4.1.3. Glands and Oral Mucosa

Beyond the nervous system, SGs are also expressed in various epithelial tissues, where their roles remain incompletely understood. Evidence from both human and animal studies consistently indicates that SG expression is spatially organized and sensitive to pathological changes, hinting at potential structural or regulatory roles.

In the pancreas, Shiga et al. [30] observed the slight expression of ζ-SG mRNA, in contrast to its strong and widespread expression in the brain. This minimal transcriptional activity could suggest that ζ-SG does not play a major role in pancreatic physiology, or that its expression is limited to a specialized subpopulation of cells, possibly within islets or ductal epithelium. While δ-SG was more broadly expressed across multiple tissues in the same study, no detailed regional or cellular localization in the pancreas was provided. This limits functional interpretation but raises questions about whether different SGs contribute to distinct cellular compartments, such as endocrine versus exocrine regions. The possibility that SGs participate in maintaining the structural integrity of pancreatic acini or islets remains open but unexplored. Given the critical role of cell polarity, adhesion, and membrane organization in secretory function, even low-level SG expression might serve stabilizing or scaffolding roles. However, without protein-level confirmation or immunolocalization data, such roles remain hypothetical.

In contrast, human studies in glandular organs such as the breast, prostate, and thyroid have provided more extensive data. Under physiological conditions, all SG subunits (α-, β-, γ-, δ-, ε-, and ζ-SG) are expressed with a uniform distribution throughout the apical, lateral, and basal surfaces of epithelial cells [27,29,36]. These patterns, confirmed by 3D reconstructions and colocalization with myoepithelial markers, suggest that SGs are integral components of epithelial cell architecture and may contribute to cell polarity, adhesion, or barrier function. Importantly, SG expression is sensitive to disease state. In healthy and pathological conditions of the breast and prostate, SG expression is markedly reduced or even lost [27,36]. This downregulation is not limited to protein localization but also involves decreased mRNA levels, as shown by RT-PCR. Similarly, in Hashimoto’s thyroiditis, SG expression in thyrocytes is significantly diminished and often completely absent in affected cells [29]. These observations raise the possibility that SG loss may not be merely a consequence of cellular dysfunction but could also contribute to epithelial disorganization, impaired intercellular signaling, or altered immune interactions. The mechanisms behind this downregulation remain uncertain. It is unclear whether SG expression in epithelial tissues is directly modulated by inflammatory signals, altered ECM composition, or hormonal control. Moreover, the functional implications of SG loss, whether it leads to impaired epithelial barrier integrity, altered mechanotransduction, or increased susceptibility to cell transformation, have yet to be addressed experimentally.

The oral mucosa provides another example of SG sensitivity to environmental and pharmacological stress. Studies in human tissue reveal that SGs are expressed in the basal and suprabasal layers of the oral epithelium, but their expression is progressively reduced in patients undergoing long-term bisphosphonate therapy, especially those with medication-related osteonecrosis of the jaw [32,35]. Interestingly, while SG expression decreases in tissue without osteonecrosis, it appears paradoxically upregulated in samples from patients with established necrosis [32], possibly reflecting a reactive or compensatory remodeling response.

This evidence points to a broader, context-dependent role for SGs in epithelial biology. Their expression appears tightly regulated under physiological conditions and dynamically modulated in response to pathological conditions. Downregulation of these proteins in pathological states suggests functional relevance, but a mechanistic understanding is lacking. Without functional studies, it remains uncertain whether SGs in these tissues form classical DGC-like complexes or participate in alternative membrane scaffolds. The co-expression of SGs with DGC components such as DG or SSPN in these tissues has not been examined. Future studies should explore whether loss of epithelial SGs plays a causal role in disease progression at the level of glandular and mucosal epithelia.

#### 4.1.4. Adipose

Emerging data suggest the relevance of SGs in adipose biology. Studies in rodents have identified a selective pattern of SG expression in white adipose tissue, particularly implicating β-, δ-, and ε-SG, alongside core components of the DGC, such as β-DG, utrophin, and SSPN [39,40]. These results suggest that adipocytes may form a non-canonical and tissue-specific DGC. Romo-Yáñez et al. [39] showed that SG transcripts are present throughout adipocyte differentiation, from preadipocytes to mature cells, in both visceral and subcutaneous depots. Notably, α- and γ-SG are consistently absent, reinforcing the idea that SG assembly is highly context-dependent and adapted to local structural and signaling needs. The persistence of β-, δ-, and ε-SG throughout all stages of adipogenesis further suggests a role in membrane stabilization or intracellular signaling, rather than a transient developmental function. In addition, Groh et al. [40] demonstrated that deletion of either β- or δ-SG leads to destabilization of the entire adipocyte SG complex, including a reduction in glycosylated α-DG, which compromises laminin binding despite the presence of the core DG protein. This disruption suggests that SGs in adipose tissue are not redundant but necessary to maintain proper DGC assembly and their interaction with the extracellular matrix. The fact that α-SG deletion does not destabilize the complex highlights a divergence from muscle biology, where α-SG is indispensable for complex integrity. These observations suggest that the adipocyte SG complex may play a role in anchoring the cytoskeleton to the extracellular matrix, regulating cell shape, or maintaining membrane stability under metabolic or mechanical stress. Additionally, the co-expression of syntrophins and dystrobrevins in differentiating adipocytes suggests possible signaling roles, although the exact pathways involved remain undefined [39].

Despite this growing body of evidence, direct functional studies on SGs in adipose physiology are lacking. It is unclear whether SG deficiencies influence adipocyte metabolism, insulin signaling, or inflammation, factors critically relevant to obesity and metabolic syndrome. Moreover, human data are absent, limiting translational interpretation.

#### 4.1.5. Other Organs

Compared to previously described tissues, SGs expression in visceral organs such as the kidney, liver, lung, spleen, and testis has been less extensively explored and remains poorly understood. Nevertheless, early transcriptomic data from rodent models indicate that certain SG subunits, particularly ε-SG and δ-SG, are expressed at varying levels across these tissues [30,38].

In particular, ε-SG mRNA was found at moderate to high levels in the kidney and lung, and at lower levels in the liver, spleen, and testis [38], suggesting a broad but non-uniform distribution. Shiga et al. [30] further reported that ζ-SG mRNA is faintly detectable in several non-neural tissues, including the liver and kidney, in contrast to its strong expression in the brain. These data suggest that while SGs are not exclusive to excitable tissues, their expression is likely tissue-specific and finely regulated. However, despite these transcript-level findings, no functional analyses or protein-level localization studies have been reported for most of these organs. The cell types on which to assess SG expression, whether epithelial, endothelial, stromal, or immune-derived, remain unidentified. Furthermore, it is unclear whether these SGs participate in classical DGC assemblies or form alternative complexes with distinct partners, as proposed for retina and adipose tissue.

This lack of data represents a significant knowledge gap. The possibility that SGs could contribute to organ-specific membrane stability, epithelial polarity, or mechanotransduction remains speculative. Moreover, whether SG expression is altered in diseases affecting these organs—such as nephropathies, hepatic fibrosis, or pulmonary hypertension—has not been explored. Given the diversity of mechanical and signaling demands across these tissues, it is plausible that SGs may perform supportive or regulatory roles that differ markedly from those observed in muscle and brain. Nonetheless, without cellular resolution, protein localization, or disease-model data, these hypotheses remain untested.

### 4.2. Focus of the Post-Translational Modifications (PTMs) of SGs: Glycosylation

Glycosylation is the most consistently reported post-translational modification of SGs across non-muscle organs. Evidence from the CNS clearly demonstrates that ζ-SG, like other SGs, undergoes N-linked glycosylation [30]. This was confirmed by molecular weight shifts following PNGase F treatment, observed not only in native brain tissue but also in cellular systems (CHO cells) where SGs were co-expressed with DGs, confirming a conserved biochemical nature. These results suggest that glycosylation is integral to the formation and stability of the SG complex. However, the functional consequences of this modification in the brain remain completely unexplored. In the retina, Fort et al. [34] confirmed the presence of glycosylated SG subunits, including β-, δ-, and ε-SG, even in the absence of dystrophin, indicating glycosylation as an independent feature from DGC integrity. However, no glycosylation-specific experiments were conducted, leaving open the question of whether SGs contribute to the formation of unique glycoprotein complexes in Müller glia or photoreceptors. In the PNS, Imamura et al. [33] indirectly confirmed the glycosylated state of SG-associated DGC proteins in Schwann cells via WGA chromatography. On the other hand, the loss of δ-SG in BIO14.6 hamsters caused destabilization of glycosylated α-DG [25]. These data suggest that SG-dependent glycosylation may modulate DGC composition and function, particularly in glial and Schwann cells.

Similarly, in adipose tissue, Groh et al. [40] showed that the absence of β- or δ-SG destabilized the glycosylated form of α-DG, impairing laminin binding. This observation suggests that SGs could indirectly control the glycosylation status of associated DGC components. However, SGs themselves were not directly analyzed for glycosylation status in this context. Despite the clear expression of SGs in epithelial and glandular tissues, no data are currently available on the glycosylation patterns of SGs. Given the role of glycosylation in adhesion and cell–matrix interactions, this represents a significant gap, especially in tissues where polarity and tight junctions are crucial.

## 5. Limitations and Gaps in the Literature

Despite growing interest in the role of SGs beyond skeletal and cardiac muscle, our understanding of their biological significance across non-muscle organs remains incompletely characterized. Much of the current knowledge stems from animal models and relies heavily on descriptive immunolocalization studies. Although these investigations have revealed consistent SG expression in various tissues, including the central and peripheral nervous systems, glands, adipose tissue, and the oral mucosa, they often lack the molecular depth and functional integration necessary to establish the physiological or pathological relevance of these findings.

One of the most pervasive limitations is the technical heterogeneity across studies. Differences in experimental protocols, including antibody specificity, tissue fixation methods, staining conditions, and imaging analysis, undermine the comparability of results. For example, the widely reported “spot-like” distribution of SGs in neurons, glia, and epithelial cells has been variably interpreted, but without standardized criteria for subcellular annotation or sufficient functional validation. The specificity of SG staining is further complicated by inconsistencies in antibody performance and the lack of rigorous controls in some studies, which raises questions about data reproducibility and the interpretation of results.

The distribution of studies across organs is highly uneven. The CNS remains the most extensively investigated, but the bulk of available evidence derives from animal models, with limited validation in human tissues. Notably, SG expression in the eye is supported by only a single study focused on the retina, underscoring a significant gap in ocular research. Glandular organs have been studied in a handful of human investigations; however, these studies are largely descriptive and limited to comparisons between normal and pathological states. Functional implications remain speculative and cross-species validation is lacking. The oral mucosa, although represented by two human studies, remains poorly understood from a mechanistic perspective. Similarly, in adipose tissue, SG expression has been documented in rodents, but the functional relevance and regulation of these complexes, especially in humans, remain unexplored. Furthermore, the molecular characterization of SGs in non-muscle contexts remains incomplete and uneven across organs. While certain PTMs, most notably N-linked glycosylation, have been confirmed in the CNS and adipose tissue, other important PTMs remain largely unclear. However, no study to date has directly tested whether SGs undergo these modifications, leaving a critical gap in understanding how membrane retention and spatial compartmentalization are regulated in diverse cellular environments.

Adding to this complexity, the functional consequences of SG expression in non-muscle organs remain largely unexplored. While data from the nervous system suggest roles in GABAergic signaling, myelin architecture, and neurovascular interactions, these remain exceptional cases. In contrast, tissues such as the thyroid, prostate, mammary gland, and oral mucosa exhibit marked SG downregulation under pathological conditions (e.g., Hashimoto’s thyroiditis, adenocarcinoma, or drug-induced osteonecrosis), yet the biological significance of these changes is unknown. Whether SG loss reflects disrupted cell–matrix adhesion, altered membrane polarity, or impaired signaling remains speculative in the absence of mechanistic or functional studies.

Finally, the lack of standardized methodological frameworks and multi-modal analyses hinders the development of a coherent model for SG function outside the muscle. Most studies fail to integrate transcriptomic, proteomic, and functional data within the same tissue, preventing meaningful cross-organ comparisons. The use of different animal models, developmental stages, and tissue processing protocols further compounds this issue, especially given the tissue-specific diversity in SG subunit composition and PTM landscapes. Future research should aim to bridge these gaps through multi-modal approaches that integrate molecular, cellular, and physiological analyses. Priorities include validating SG expression and localization in human tissues, characterizing their post-translational modifications and binding partners across organ systems, and investigating their roles in maintaining tissue architecture and function under both physiological and pathological conditions. Such comprehensive studies are essential for clarifying the roles of SGs in non-muscle organs and for evaluating their potential as biomarkers or therapeutic targets beyond the muscular system.

## 6. Conclusions

This scoping review emphasizes that SGs, classified as muscle-specific proteins, are widely expressed in non-muscle organs and tissues with heterogeneous expression patterns and subcellular localizations, suggesting novel roles in synaptic organization, cell adhesion, and neurovascular integrity. Nevertheless, considerable gaps exist concerning the molecular composition and functional implications of SG complexes in these tissues. Although evidence of conserved molecular features, such as N-linked glycosylation and interactions with scaffold proteins, is emerging, the composition, regulation, and mechanistic roles of SG complexes in non-muscle contexts remain incompletely defined. Future research should aim to elucidate these aspects using integrated molecular, cellular, and functional approaches. Advanced techniques such as proteomics and gene editing may reveal novel interactors and tissue-specific regulatory mechanisms. To support a broader conceptual framework for these emerging functions, we tentatively propose the functional term *Anastasi Linkage Proteins* (ALPs) to describe SG-containing complexes in non-muscle contexts. This terminology, honoring Professor Giuseppe Pio Anastasi’s contributions, is not intended as a formal genetic reclassification, but rather to support a conceptual shift toward their recognition as widely distributed, functionally diverse membrane complexes.

## Figures and Tables

**Table 5:** Overview of the studies included for gland organs.

Study (Authors, Year)	Study Design	Tissue/Organ/Cell Types Examined	Types of SG Proteins Studied	Species/Models
Shiga et al. (2006) [30]	Immunofluorescence and molecular (RT-PCR and immunoprecipitation) study	Pancreas	δ, ζ	Mouse
Arco et al. (2012) [27]	Immunohistochemical and molecular study (RT-PCR)	Glandular breast (epithelial and myoepithelial cells)	α, β, γ, δ, ε	Human
Cutroneo et al. (2014) [36]	Immunohistochemical and molecular (RT-PCR) study	Prostate gland (epithelial and myoepithelial cells)	α, β, γ, δ, ε	
Favaloro et al. (2022) [29]	Immunofluorescence study	Thyroid	α, β, γ, δ, ε, ζ	

**Table 6:** Overview of the studies included for the oral mucosa.

Study (Authors, Year)	Study Design	Tissue/Organ/Cell Types Examined	Types of SG Proteins Studied	Species/Models
Nastro Siniscalchi et al. (2010) [32]	Immunohistochemical study	Gingival epithelium	β, γ, ε	Human
De Ponte et al. (2013) [35]	Immunohistochemical study	Gingival epithelium	α, γ, ε	

**Table 7:** Overview of the studies included for the adipose organ.

Study (Authors, Year)	Study Design	Tissue/Organ/Cell Types Examined	Types of SG Proteins Studied	Species/Models
Groh et al. (2009) [40]	Molecular study (RT-PCR and Western blot)	White adipocytes	α, β, δ	Mouse
Romo-Yáñez et al. (2011) [39]	Molecular study (RT-PCR and Western blot)	Adipose tissue (differentiation adipocyte)	β, δ, ε	Rat

**Table 8:** Overview of the studies included for other organs.

Study (Authors, Year)	Study Design	Tissue/Organ/Cell Types Examined	Types of SG Proteins Studied	Species/Models
Xiao and LeDoux (2003) [38]	Molecular study (Northern analysis, RT-PCR and ISH)	Liver Kidney Lung Spleen Testis	ε	Rat
Shiga et al. (2006) [30]	Immunofluorescence and molecular (RT-PCR and immunoprecipitation) study	Kidney Liver Lung Spleen	δ, γ, ζ	Mouse

## Data Availability

All data are contained within the article.

## References

[1] Durbeej M., Campbell K.P. (1999). Biochemical Characterization of the Epithelial Dystroglycan Complex. J. Biol. Chem..

[2] Matsumura K., Saito F., Yamada H., Hase A., Sunada Y., Shimizu T. (1999). Sarcoglycan Complex: A Muscular Supporter of Dystroglycan-Dystrophin Interplay?. Cell. Mol. Biol..

[3] Noguchi S., Wakabayashi E., Imamura M., Yoshida M., Ozawa E. (2000). Formation of Sarcoglycan Complex with Differentiation in Cultured Myocytes. Eur. J. Biochem..

[4] Younus M., Ahmad F., Malik E., Bilal M., Kausar M., Abbas S., Shaheen S., Kakar M.U., Alfadhel M., Umair M. (2019). SGCD Homozygous Nonsense Mutation (p.Arg97∗) Causing Limb-Girdle Muscular Dystrophy Type 2F (LGMD2F) in a Consanguineous Family, a Case Report. Front. Genet..

[5] Xiao H.F., Gong S.Z., Zhao Y., LeDoux M.S. (2004). Developmental Expression of Rat TorsinA Transcript and Protein. Dev. Brain Res..

[6] Wolburg H., Wolburg-Buchholz K., Fallier-Becker P., Noell S., Mack A.F., Kwang W.J. (2011). Structure and Functions of Aquaporin-4-Based Orthogonal Arrays of Particles. International Review of Cell and Molecular Biology.

[7] Cirak S., Arechavala-Gomeza V., Guglieri M., Feng L., Torelli S., Anthony K., Abbs S., Garralda M.E., Bourke J., Wells D.J. (2011). Exon Skipping and Dystrophin Restoration in Patients with Duchenne Muscular Dystrophy after Systemic Phosphorodiamidate Morpholino Oligomer Treatment: An Open-Label, Phase 2, Dose-Escalation Study. Lancet.

[8] Chawla G., Lin C.-H., Han A., Shiue L., Ares M.J., Black D.L. (2009). Sam68 Regulates a Set of Alternatively Spliced Exons during Neurogenesis. Mol. Cell. Biol..

[9] Henry M.D., Campbell K.P. (1996). Dystroglycan: An Extracellular Matrix Receptor Linked to the Cytoskeleton. Curr. Opin. Cell Biol..

[10] Straub V., Campbell K.P. (1997). Muscular Dystrophies and the Dystrophin-Glycoprotein Complex. Curr. Opin. Neurol..

[11] Ceccarini M., Grasso M., Veroni C., Gambara G., Artegiani B., Macchia G., Ramoni C., Torreri P., Mallozzi C., Petrucci T.C. (2007). Association of Dystrobrevin and Regulatory Subunit of Protein Kinase A: A New Role for Dystrobrevin as a Scaffold for Signaling Proteins. J. Mol. Biol..

[12] Straub V., Ettinger A.J., Durbeej M., Venzke D.P., Cutshall S., Sanes J.R., Campbell K.P. (1999). Epsilon-Sarcoglycan Replaces Alpha-Sarcoglycan in Smooth Muscle to Form a Unique Dystrophin-Glycoprotein Complex. J. Biol. Chem..

[13] Wheeler M.T., McNally E.M. (2003). Sarcoglycans in Vascular Smooth and Striated Muscle. Trends Cardiovasc. Med..

[14] Wheeler M.T., Zarnegar S., McNally E.M. (2002). Zeta-Sarcoglycan, a Novel Component of the Sarcoglycan Complex, Is Reduced in Muscular Dystrophy. Hum. Mol. Genet..

[15] Shi W., Chen Z., Schottenfeld J., Stahl R.C., Kunkel L.M., Chan Y.-M. (2004). Specific Assembly Pathway of Sarcoglycans Is Dependent on Beta- and Delta-Sarcoglycan. Muscle Nerve.

[16] Chan P., Gonzalez-Maeso J., Ruf F., Bishop D.F., Hof P.R., Sealfon S.C. (2005). Epsilon-Sarcoglycan Immunoreactivity and MRNA Expression in Mouse Brain. J. Comp. Neurol..

[17] Chakrabarty B., Sharma M.C., Gulati S., Kabra M., Pandey R.M., Sarkar C. (2014). Skin Biopsy: A New Tool to Diagnose Sarcoglycanopathy. J. Child Neurol..

[18] Arena S., Favaloro A., Cutroneo G., Consolo A., Arena F., Anastasi G., Di Benedetto V. (2008). Sarcoglycan Subcomplex Expression in Refluxing Ureteral Endings. J. Urol..

[19] Arena S., Cutroneo G., Favaloro A., Sinatra M.T., Trimarchi F., Scarvaglieri S., Mallamace A., Arena F., Anastasi G., Di Benedetto V. (2010). Abnormal Distribution of Sarcoglycan Subcomplex in Colonic Smooth Muscle Cells of Aganglionic Bowel. Int. J. Mol. Med..

[20] Anastasi G., Cutroneo G., Sidoti A., Rinaldi C., Bruschetta D., Rizzo G., D’Angelo R., Tarone G., Amato A., Favaloro A. (2007). Sarcoglycan Subcomplex Expression in Normal Human Smooth Muscle. J. Histochem. Cytochem..

[21] Muni-Lofra R., Juanola-Mayos E., Schiava M., Moat D., Elseed M.A., Michel-Sodhi J., Harris E., McCallum M., Moore U., Richardson M.T. (2023). Longitudinal Analysis of Respiratory Function of Different Types of Limb Girdle Muscular Dystrophies Reveals Independent Trajectories. Neurol. Genet..

[22] Li J., Liu Y., Li Q., Huang X., Zhou D., Xu H., Zhao F., Mi X., Wang R., Jia F. (2020). Mutation in Ε-Sarcoglycan Induces a Myoclonus-Dystonia Syndrome-Like Movement Disorder in Mice. Neurosci. Bull..

[23] Tricco A.C., Lillie E., Zarin W., O’Brien K.K., Colquhoun H., Levac D., Moher D., Peters M.D.J., Horsley T., Weeks L. (2018). PRISMA Extension for Scoping Reviews (PRISMA-ScR): Checklist and Explanation. Ann. Intern. Med..

[24] Vermiglio G., Runci M., Scibilia A., Biasini F., Cutroneo G. (2012). Preliminary Study on Sarcoglycan Sub-Complex in Rat Cerebral and Cerebellar Cortex. Ital. J. Anat. Embryol..

[25] Cai H., Erdman R.A., Zweier L., Chen J., Shaw J.H., Baylor K.A., Stecker M.M., Carey D.J., Chan Y.M. (2007). The Sarcoglycan Complex in Schwann Cells and Its Role in Myelin Stability. Exp. Neurol..

[26] Boulay A., Saubaméa B., Cisternino S., Mignon V., Mazeraud A., Jourdren L., Blugeon C., Cohen-Salmon M. (2015). The Sarcoglycan Complex Is Expressed in the Cerebrovascular System and Is Specifically Regulated by Astroglial Cx30 Channels. Front. Cell. Neurosci..

[27] Arco A., Favaloro A., Gioffrè M., Santoro G. (2012). Sarcoglycans in the Normal and Pathological Breast Tissue of Humans: An Immunohistochemical and Molecular Study. Cells Tissues Organs.

[28] Anastasi G., Tomasello F., Di Mauro D., Cutroneo G., Favaloro A., Conti A., Ruggeri A., Rinaldi C., Trimarchi F. (2012). Expression of Sarcoglycans in the Human Cerebral Cortex: An Immunohistochemical and Molecular Study. Cells Tissues Organs.

[29] Favaloro A., Rizzo G., Santoro G., Pergolizzi S., Furci A., Centofanti A., Cutroneo G. (2022). Sarcoglycans and Integrins in Human Thyrocytes: An Immunofluorescence Study. Ital. J. Anat. Embryol..

[30] Shiga K., Yoshioka H., Matsumiya T., Kimura I., Takeda S., Imamura M. (2006). Zeta-Sarcoglycan Is a Functional Homologue of Gamma-Sarcoglycan in the Formation of the Sarcoglycan Complex. Exp. Cell Res..

[31] Rizzo G., Di Mauro D., Cutroneo G., Schembri-Wismayer P., Brunetto D., Spoto C., Vermiglio G., Centofanti A., Favaloro A. (2018). An Immunofluorescence Study About Staining Pattern Variability of Sarcoglycans in Rat’s Cerebral and Cerebellar Cortex. Eur. J. Exp. Biol..

[32] Nastro Siniscalchi E., Cutroneo G., Catalfamo L., Santoro G., Allegra A., Oteri G., Cicciù D., Alonci A., Penna G., Musolino C. (2010). Immunohistochemical Evaluation of Sarcoglycans and Integrins in Gingival Epithelium of Multiple Myeloma Patients with Bisphosphonate-Induced Osteonecrosis of the Jaw. Oncol. Rep..

[33] Imamura M., Araishi K., Noguchi S., Ozawa E. (2000). A Sarcoglycan-Dystroglycan Complex Anchors Dp116 and Utrophin in the Peripheral Nervous System. Hum. Mol. Genet..

[34] Fort P., Estrada F.-J., Bordais A., Mornet D., Sahel J.-A., Picaud S., Vargas H.R., Coral-Vázquez R.M., Rendon A. (2005). The Sarcoglycan-Sarcospan Complex Localization in Mouse Retina Is Independent from Dystrophins. Neurosci. Res..

[35] De Ponte F.S., Favaloro A., Siniscalchi E.N., Centofanti A., Runci M., Cutroneo G., Catalfamo L. (2013). Sarcoglycans and Integrins in Bisphosphonate Treatment: Immunohistochemical and Scanning Electron Microscopy Study. Oncol. Rep..

[36] Cutroneo G., Bramanti P., Favaloro A., Anastasi G., Trimarchi F., Di Mauro D., Rinaldi C., Speciale F., Inferrera A., Santoro G. (2014). Sarcoglycan Complex in Human Normal and Pathological Prostatic Tissue: An Immunohistochemical and RT-PCR Study. Anat. Rec..

[37] Cutroneo G., Bramanti P., Anastasi G., Bruschetta D., Favaloro A., Vermiglio G., Trimarchi F., Di Mauro D., Rizzo G. (2015). Sarcoglycans and Gaba(a) Receptors in Rat Central Nervous System: An Immunohistochemical Study. Ital. J. Anat. Embryol..

[38] Xiao J., LeDoux M.S. (2003). Cloning, Developmental Regulation and Neural Localization of Rat Epsilon-Sarcoglycan. Brain Res. Mol. Brain Res..

[39] Romo-Yáñez J., Montañez C., Salazar-Olivo L.A. (2011). Dystrophins and DAPs Are Expressed in Adipose Tissue and Are Regulated by Adipogenesis and Extracellular Matrix. Biochem. Biophys. Res. Commun..

[40] Groh S., Zong H., Goddeeris M.M., Lebakken C.S., Venzke D., Pessin J.E., Campbell K.P. (2009). Sarcoglycan Complex: Implications for Metabolic Defects in Muscular Dystrophies. J. Biol. Chem..

[41] Cazurro-Gutiérrez A., Marcé-Grau A., Correa-Vela M., Salazar A., Vanegas M.I., Macaya A., Bayés À., Pérez-Dueñas B. (2021). ε-Sarcoglycan: Unraveling the Myoclonus-Dystonia Gene. Mol. Neurobiol..

